# Fire Activity and Severity in the Western US Vary along Proxy Gradients Representing Fuel Amount and Fuel Moisture

**DOI:** 10.1371/journal.pone.0099699

**Published:** 2014-06-18

**Authors:** Sean A. Parks, Marc-André Parisien, Carol Miller, Solomon Z. Dobrowski

**Affiliations:** 1 Aldo Leopold Wilderness Research Institute, Rocky Mountain Research Station, USDA Forest Service, Missoula, Montana, United States of America; 2 Northern Forestry Centre, Canadian Forest Service, Natural Resources Canada, Edmonton, Alberta, Canada; 3 Department of Forest Management, College of Forestry and Conservation, University of Montana, Missoula, Montana, United States of America; US Geological Survey, United States of America

## Abstract

Numerous theoretical and empirical studies have shown that wildfire activity (e.g., area burned) at regional to global scales may be limited at the extremes of environmental gradients such as productivity or moisture. Fire activity, however, represents only one component of the fire regime, and no studies to date have characterized *fire severity* along such gradients. Given the importance of fire severity in dictating ecological response to fire, this is a considerable knowledge gap. For the western US, we quantify relationships between climate and the fire regime by empirically describing both fire activity and severity along two climatic water balance gradients, actual evapotranspiration (AET) and water deficit (WD), that can be considered proxies for fuel amount and fuel moisture, respectively. We also concurrently summarize fire activity and severity among ecoregions, providing an empirically based description of the geographic distribution of fire regimes. Our results show that fire activity in the western US increases with fuel amount (represented by AET) but has a unimodal (i.e., humped) relationship with fuel moisture (represented by WD); fire severity increases with fuel amount and fuel moisture. The explicit links between fire regime components and physical environmental gradients suggest that multivariable statistical models can be generated to produce an empirically based fire regime map for the western US. Such models will potentially enable researchers to anticipate climate-mediated changes in fire recurrence and its impacts based on gridded spatial data representing future climate scenarios.

## Introduction

Fire is a ubiquitous ecosystem process across the globe. The concept of the fire regime has been used to describe the role of fire in an ecosystem in terms of its spatial-temporal patterns and ecosystem impacts [1]. As such, maps of fire regimes, in terms of fire frequency and severity, have been produced [2]. These maps not only allow geographic comparisons of fire regime components [3], but are also a necessary first step for describing shifts in fire regimes resulting from factors such as climate change [4], fire suppression [5], and invasive species [6]. Though the importance of mapping fire regimes is long acknowledged [7], [8], efforts to date have been largely qualitative and thus have been unable to make direct linkages to factors driving both fire activity and severity (but see [9]).

Recently, however, linkages between environmental gradients and fire activity have been identified. Several studies have shown that wildland fires across the globe tend to avoid environmental extremes of productivity and moisture [10]–[13]. For example, in very dry areas such as deserts, fire activity (i.e., fire occurrence and area burned) is limited by a lack of biomass [14]. Conversely, in the wettest places on Earth (e.g., rainforests), there is ample biomass but fire activity is limited because climate conditions promoting combustion are rare [15]. To date, studies of the geographic distribution of fire, or pyrogeography, have focused more on patterns of *fire activity* (e.g., [16]–[18]) than on explaining patterns of *fire severity* (but see [19], [20]). Given that fire severity – a measure of ecosystem change – can strongly dictate the response of biological communities to fire [21]–[23], an understanding of its environmental controls is a prerequisite for understanding the role of fire in ecosystems. Furthermore, without an understanding of these controls, modeling and prediction of climate-mediated changes in fire regimes is tenuous.

The seminal biome classification of Whittaker [24] exemplifies the utility of representing large scale ecological features along an energy-moisture biplot (temperature and precipitation in this case). As a refinement of Whittaker’s framework, Stephenson [25] developed a bivariate representation of North American’s main vegetation types using actual evapotranspiration (AET) and climatic water deficit (WD; potential evapotranspiration minus AET), as measures of productivity and drought, respectively. Specifically, AET represents a measure of available moisture to plants, whereas WD is a measure of unmet atmospheric demand for water (i.e., how much water could be evaporated and transpired were it available) [25]. Though related to precipitation and temperature, AET and WD take into account the concurrent availability and demand for moisture and therefore better represent environmental controls on ecosystems. As such, these metrics have been shown to be strong predictors of plant physiognomic types [25] and tree species distributions [26].

Recent studies have shown that water balance metrics such as AET and WD are predictive of annual to decadal fire activity [27], [28]. AET is strongly correlated with biomass production [29],[30], and as such, it is a suitable proxy for fuel amount in many ecosystems. WD is a measure of absolute drought and should reflect the moisture status and flammability of live [31] and large-diameter dead fuels [28]. Previous research has suggested a unimodal (i.e., humped) response of fire activity to fuel amount (e.g., [11]) and fuel moisture [32]; that is, less fire occurs at the extremes of these gradients than at intermediate levels. Less clearly understood are the relationships between severity and these gradients. Some studies have suggested that fire severity depends upon amount of biomass [33] and therefore would be expected to increase with AET; the relationship between fire severity and fuel moisture, however, is unknown.

We seek to better understand how contemporary fire regimes across the western US vary along macroscale environmental gradients. Our first objective is to examine how fire activity and fire severity each vary as a function of proxy gradients for fuel amount and fuel moisture (represented by AET and WD, respectively). Our second objective is to present an empirically based characterization of fire regimes that includes both fire activity and fire severity. Specifically, we characterize fire activity and severity by ecoregion, thus quantitatively describing ecoregions within a pyrogeographic context that includes more than one fire regime component. Because anthropogenic activities can obscure fire-climate relationships [34], we focus our analysis on protected areas. This study explicitly links fire regime components with physical environmental gradients, and therefore is an important first step toward forecasting and mitigation of projected changes in fire regimes.

## Methods

### Data

Fire data for the 1984–2010 period were obtained from the Monitoring Trends in Burn Severity (MTBS) project [35], which has mapped fire perimeters and fire severity for all fires ≥400 ha in the western US. We define fire severity as the degree of fire-induced change to vegetation and soils as measured by the delta normalized burn ratio (dNBR), a satellite-inferred index that differences pre- and post-fire Landsat images [36]. Raw dNBR values were adjusted to account for differences due to phenology or precipitation between the pre- and post-fire images by subtracting the average dNBR of pixels outside the burn perimeter; this adjustment can be important when comparing dNBR among fires [37]. As dNBR values increase, there is generally an increase in char and scorched/blackened vegetation and a decrease in moisture content and vegetative cover [36]. The dNBR is strongly associated with field-assessed measures of fire severity over a broad range of ecosystem types [38]–[41]. In very limited cases, fire perimeters had no associated dNBR; this was generally the case for fires that occurred in 1984 and pre-fire Landsat TM data were unavailable.

Gridded annual AET and WD data (30 arc-second resolution; ∼800 m) were obtained from Dobrowski et al. [42] and were derived from a modified Thornthwaite-Mather soil water balance model. The model was run on a monthly time step and accounts for atmospheric demand (potential evapotranspiration – calculated using the Penman-Monteith equation [43]), soil water storage, and includes the effect of temperature and radiation on snow hydrology via a snowmelt model. Annual values for AET and WD were averaged for each pixel over the 1984–2010 time period, corresponding to the fire data used in this study. Low values of AET represent areas with low potential productivity and thus limited fuel amount, whereas high values indicate the potential for high fuel amounts. Low values of WD indicate that water is less limiting (i.e., high fuel moisture), whereas high values indicate severe water limitation. Given that these water balance metrics are correlated in this study (Pearsons’s *r*>0.75), it may be difficult to disentangle the unique contribution of each term.

### Analysis

**Figure 1:**
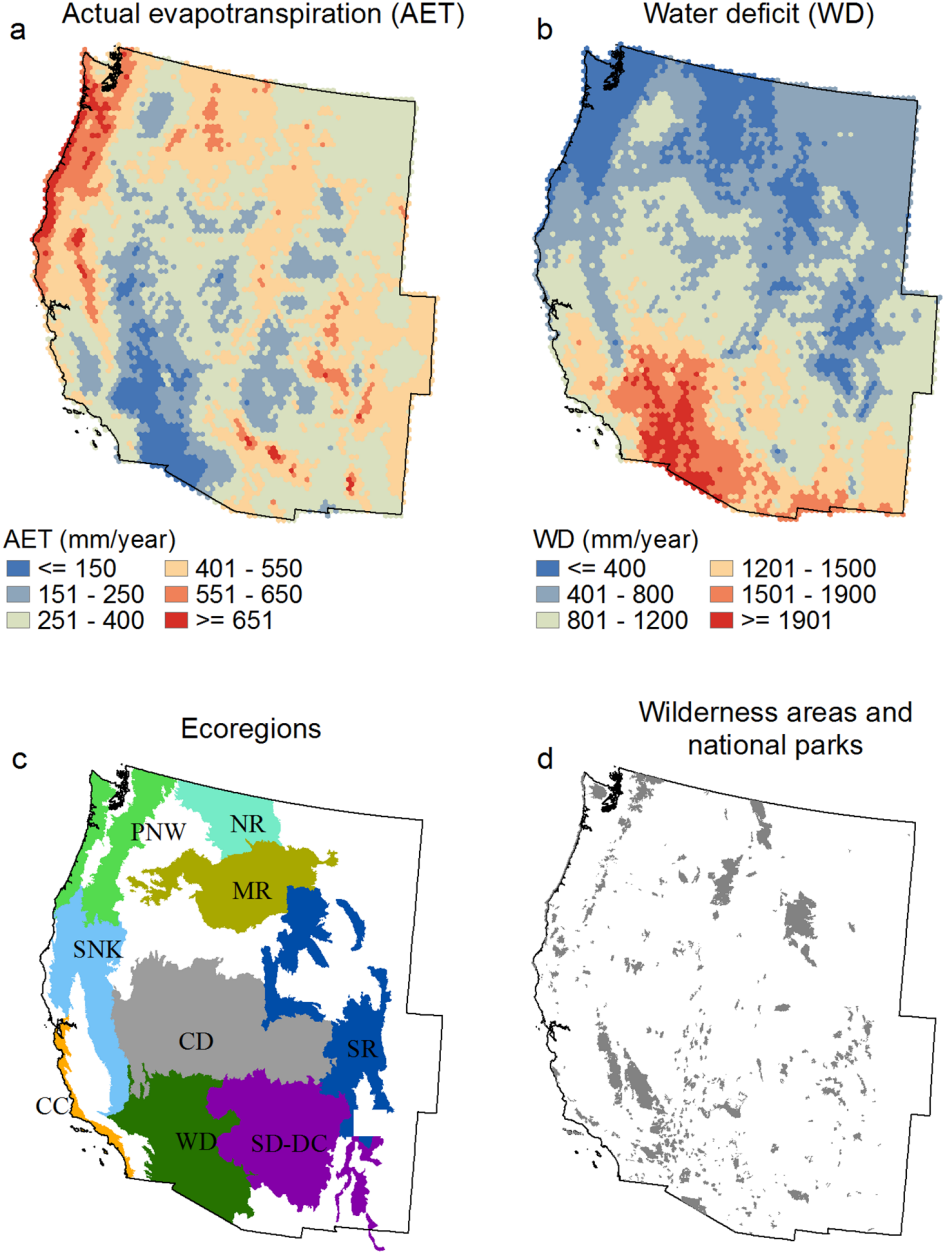
Mean actual evapotranspiration (AET) (a) and water deficit (WD) (b) per 50,000 ha hexel for the western US from 1984–2010. Also shown are the ecoregions (USDA Forest Service, 2007) we analyzed (c): California chaparral (CC), cold desert (CD), middle Rocky Mountains (MR), northern Rocky Mountains (NR), Pacific Northwest (PNW), semi desert – dry conifer (SD-DC), Sierra Nevada and Klamath (SNK), southern Rocky Mountains (SR), warm desert (WD). Cold desert and semi desert – dry conifer are both the result of merging two ecoregions; all ecoregions were renamed for easier interpretation. Areas in white represent ecoregions that did not contain enough fire and/or wilderness and national park area to be included in this study (see Methods). Locations of wilderness areas and national parks in the western US (d).

The western US was partitioned into 50,000-ha (500 km^2^) hexels in which fire activity and severity were summarized. Although the spatial resolution of fire-related analyses has been shown to influence outcomes [44], [45], this hexel size was deemed a reasonable compromise for capturing variability among sample units while maintaining an adequate sample size for analysis, especially given the inherent spatial variability associated with the relatively short fire record we analyzed (1984–2010). Fire activity was calculated as the proportion of each hexel (excluding nonfuel, such as barren, ice/snow, etc. [3]) that burned from 1984 to 2010; this proportion was subsequently square root transformed to homogenize variance in model residuals. Fire severity was calculated by averaging the dNBR of all pixels of all fires that intersected each hexel; pixels classified as nonfuel were excluded in the calculation of the mean. AET and WD were also aggregated to the hexel scale (Fig. 1).

To characterize how fire activity varies along gradients in fuel amount and fuel moisture, we built bivariate statistical models of area burned as a function of AET and WD; similar models were produced with dNBR to characterize how fire severity varies along these gradients. The two models of area burned were generated using generalized linear models (family = quasibinomial) with a quadratic term (i.e., 2^nd^ order polynomial) for AET and WD. dNBR was assessed as a function of AET using an ordinary least squares (ols) model, whereas dNBR was related to WD using a nonlinear exponential function. All model fits (except the dNBR vs. AET model) were evaluated using the coefficient of determination (denoted here as R^2^), which is the r^2^ of a linear regression between observed and predicted values (cf. [39], [46]); the dNBR vs. AET model was evaluated using ols r^2^. In our initial data exploration, we also evaluated dNBR as a function of AET using an exponential function, but this resulted in a decreased model strength compared to the linear fit. To reduce the effect of human infrastructure that can disrupt the spread of fires (e.g., roads) and vegetation management activities that can alter fuel structures (e.g., silviculture), we limited this analysis to hexels comprising ≥80% designated wilderness and national park; a total of 153 hexels met this criterion for the fire activity models. An additional criterion was added for the evaluation of fire severity to limit variability in mean dNBR values that are associated with small sample sizes (cf. [47]): at least 400 ha had to have burned within a hexel from 1984–2010. A total of 99 hexels met this additional criterion for the fire severity models.

To place our data and findings into a broader biogeographic context, we summarized the hexels (using the previously described subset based on minimum of 80% wilderness and national park) in each ‘ecological section’ (hereafter ecoregion) as defined by ECOMAP [48] (Fig. 1). We plotted each ecoregion along axes of AET and WD by averaging the values among hexels in each ecoregion. We similarly plotted each ecoregion along axes of area burned and dNBR, thereby providing a broad-scale concurrent characterization of two components of the fire regime. All ecoregions were renamed and two pairs of two ecoregions were merged to ‘cold desert’ and ‘semi desert – dry conifer’ for easier interpretation.

Although not a primary objective of our study, we were able to evaluate how changing the sampling criteria for inclusion of hexels into the models affected the strength of the relationships between the fire characteristics (area burned and dNBR) and the climatic water balance metrics (AET and WD). For each of the four relationships, we built 20 models with different minimum requirements for amount of within-hexel designated wilderness and national park, ranging from zero to 95% (5% increments).

## Results

**Figure 2:**
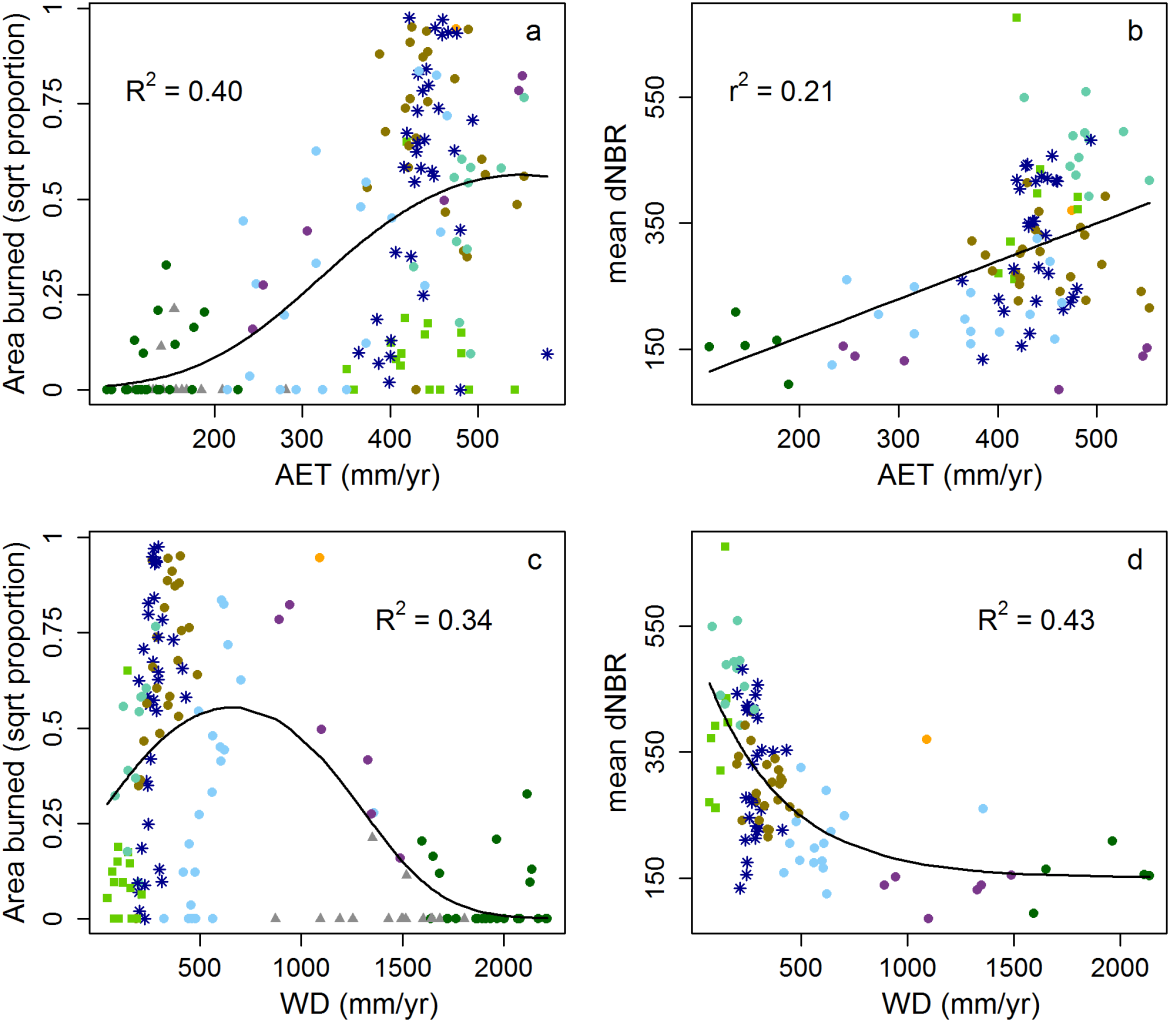
Area burned plotted against AET (a) and WD (b). Mean dNBR plotted against AET (c) and WD (d). Fitted lines are generated by models of each relationship (see Methods); the strength of each model is reported (R^2^ or r^2^). Symbol colors correspond to the ecoregion in which it is located (Fig. 1).

The relationships of area burned and dNBR to AET and WD are moderately strong. Area burned increases with AET (R^2^ = 0.40), as does dNBR (r^2^ = 0.21) (Fig. 2a, 2b). Area burned and WD have a unimodal relationship (R^2^ = 0.34), whereas dNBR decreases with WD (R^2^ = 0.43) (Fig. 2c, 2d).

**Figure 3:**
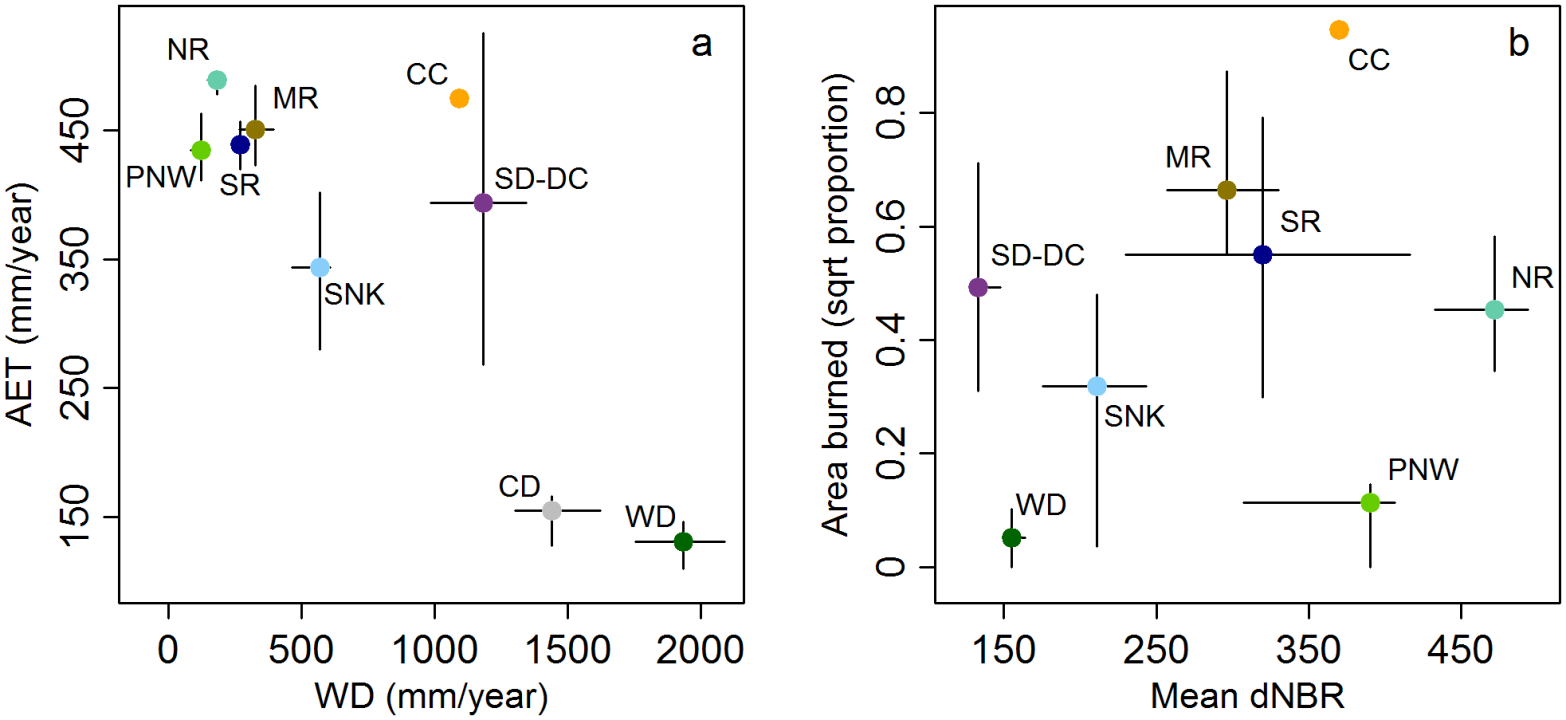
AET and WD averaged across hexels in each ecoregion [48] (a). Area burned and dNBR averaged across hexels in each ecoregion (b). Although the cold desert ecoregion did experience some fire within the hexels we analyzed (see Fig. 2), there were no dNBR data for this ecoregion (see Methods); therefore, this ecoregion is not included in the plot on the right (b). Vertical and horizontal lines represent the middle 50 percent (25^th^ to 75^th^ percentile) of the hexel values in each ecoregion.

Broad ecoregion-level biogeographic patterns are revealed in the biplot of AET and WD (Fig. 3a); extreme differences in both AET and WD among contrasted ecoregions, such as the warm desert (WD) and Pacific Northwest (PNW), are evident. In general, ecoregions occupy distinct portions of the AET and WD bi-dimensional space, but the Pacific Northwest and all Rocky Mountain ecoregions are relatively tightly grouped (Fig. 3a). Ecoregional differences in fire regime characteristics are also apparent in the biplot of area burned and dNBR (Fig. 3b). The tight grouping of some ecoregions seen in the biplot of AET and WD (Fig. 3a) is not seen in the biplot of fire regime characteristics (Fig. 3b).

**Figure 4:**
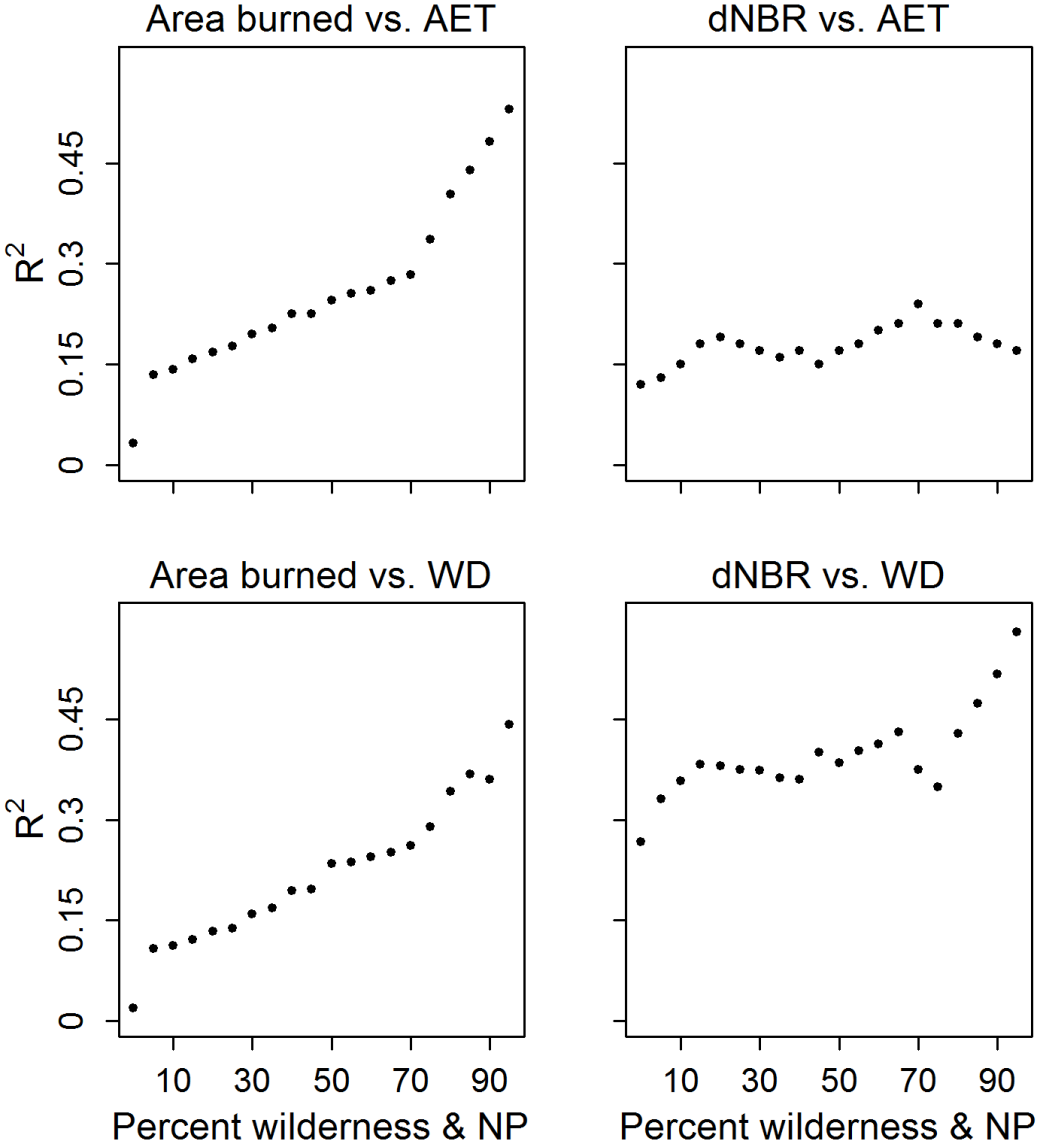
Strength of the relationship (R^2^ for all models except the dNBR vs. AET model, which is evaluated with the r^2^; see Methods) for all models as a function of increasing percentage of wilderness and national park in each hexel.

The relationships generally strengthened as we increased the requisite percent of wilderness and national park for inclusion in the models (Fig. 4). In one of the four cases (area burned vs. AET), the R^2^ increased from 0.03 to 0.53 when the minimum within-hexel percent wilderness and national park increased from 0% to 95%, respectively.

## Discussion

Fire activity increased with AET, the proxy we used for fuel amount; the unimodal relationship that previous theoretical and empirical studies have reported was not seen here [11]–[13]. This is likely because our study was limited to the western US, and therefore, we evaluated a truncated range of AET (the highest AET values in the US are located in the Southeast). Had we been able to include ecosystems with higher AET values in our analysis, we expect we would have seen a limitation on fire activity at the extreme upper values of AET. Fire activity had a unimodal relationship with fuel moisture, represented by WD, concurring with the theoretical model described by Pausas and Bradstock [32]. Consistent with the “varying constraints hypothesis” posited by Krawchuk and Moritz [11], areas in ecoregions with the highest fuel moisture (lowest WD) (i.e., Pacific Northwest) experienced relatively low fire activity because, despite being biomass rich, they are rarely dry enough to burn. At the other end of the gradient, fire activity in ecoregions with low fuel moisture (i.e., warm desert) was limited because low productivity limits the amount of biomass that can burn.

Fire severity increased with fuel amount, providing support to the idea that fire severity is limited by available biomass [33], [49]. The relationship between fire severity and fuel moisture distinctly showed an exponential decrease in fire severity with increasing WD (see Fig. 2d). This is a somewhat counterintuitive and unexpected result because lower fuel moisture has been found to result in higher severity fires [20], [50], as drought-stressed trees may be more susceptible to damage by fire and low fuel moisture increases the flammability of fuels. The apparent discrepancy likely involves key differences and interpretations between analyses that evaluate the influence of short-term (e.g., annual) variability (e.g., [20], [50]) and those that evaluate long-term averages (our study). For example, Parisien et al. [51] found that the factors controlling fire activity were strikingly different when a statistical model was built incorporating annual variability compared to one using long-term (∼30 years) averages. In our study, we evaluated fairly long-term fuel moisture (i.e., averaged over 26 years), as measured by WD, which doesn’t necessarily reflect the short-term moisture conditions under which fires actually occur. Consequently, the influence of fuel moisture on fire severity seen in this study may be due to its long-term climatic influence on the establishment and perpetuation of forest types that are adapted to particular moisture conditions; those forest types in turn influence fire severity. For example, on those occasions when moist forest types experience fire (i.e., unusually dry years), they tend to burn at high severity due to their high tree density and abundance of ladder fuels [52]. Nevertheless, the aforementioned correlation between our proxies for fuel amount and moisture (AET and WD, respectively) cannot be ignored and, consequently, completely disentangling their influence is challenging.

Broad biogeographic differences among ecoregions were evident when they were partitioned in terms of AET (fuel amount) and WD (fuel moisture). The concurrent characterization of fire activity and severity also clearly distinguished ecoregions. For example, the warm desert ecoregion experienced low fire activity and severity, whereas the Pacific Northwest ecoregion experienced low fire activity but high fire severity (Fig. 3b). However, ecoregions that were tightly grouped in terms of AET and WD (Pacific Northwest and all Rocky Mountain ecoregions) (Fig. 3a) did not exhibit a similar grouping of fire regime characteristics (Fig. 3b). This discrepancy could be due to difficulties associated with describing the fire regime using only AET and WD or the relatively short fire record that we analyzed, which potentially over- or under-emphasizes fire activity for certain regions (e.g., 1988 Yellowstone fires) [53]; other factors such as macroscale spatial variability in ignition sources [54] and fire suppression effectiveness could also be responsible. Ultimately, however, aggregating hexels by ecoregion may oversimplify complex fire regime dynamics and their effects on ecosystems.

Changes in fire activity and severity are certain to occur as water balance metrics shift in concert with a changing climate, perhaps in complex and unexpected ways [55]. The temporal covariance between energy and moisture availability will play a key role in determining how fuel amount and fuel moisture are geographically distributed in the future, with clear implications for pyrogeography. The complex interplay between climate and the seasonal timing of water and energy availability illustrates the need for mechanistic bioclimatic predictors that can account for these contingencies [25]. The framework developed in this study is an excellent starting point for predicting fire regime shifts under a changing climate though we acknowledge that climate-induced changes in fire regimes will likely have strong feedbacks with the dominant vegetation and ecosystem processes in any given area [56].

We focused this study on wilderness areas and national parks in the western US, where anthropogenic influences (e.g., forest management) are minimal relative to unprotected lands. One consequence of our focus on protected areas is that the fairly low number of hexels available to use in our models of area burned and fire severity represent only 2.5% and 1.6% of the land area in the western US, respectively. As such, the relationships we derived from these relatively unmanaged, natural lands may not well represent the majority of the western US; indeed, the relationships between fire and climate clearly weaken as the human footprint increases (see Fig. 4). Archibald et al. [34] noted a similar finding in the African savannas, in that annual climatic variability was strongly associated with large fire occurrence in areas of low human impact but not in human-dominated areas. Although the hexels we used admittedly represent a small proportion of the western US, the data we analyzed provide valuable insight regarding the “natural” relationships between fire and climate. Consequently, our approach can potentially be used to identify areas with disrupted fire regimes and in need of restoration treatments [57].

Although we used a single metric (mean dNBR) as a convenient measure of ecosystem change, fire severity is the result of many complex physical and ecological factors that are difficult to represent with simplistic measures such as dNBR. For example, lodgepole pine forests experience high-severity fire regimes that favor the regeneration and perpetuation of lodgepole pine on a site, because, as fire often kills most or all of the trees, it also opens their serotinous cones [58], [59]. In this example, although dNBR would indicate high severity fire, the change to the ecosystem would be considered far less drastic because lodgepole pine will remain the dominant tree species. As such, our analyses do not address if fire severity is within its historical range of variability [60] for any given vegetation community. Furthermore, dNBR in some ecosystems such as semi-arid shrublands (low AET and high WD) may be relatively low even though the degree of fire-induced change may be very high due to factors such as high rates of post-fire erosion [61] and conversion of perennial woodlands to annual grasslands [62]. Consequently, the relationships reported in this study could be different were other aspects of fire severity (those that cannot be measured with satellites) evaluated. It has also been argued that *relative* metrics of burn severity (i.e., those that measure change relative to the amount of pre-fire vegetation) are more appropriate than absolute metrics (i.e., dNBR) on sites where pre-fire vegetation is low [63], [64]. The reasoning for this argument is that dNBR values will be lower on sparsely vegetated sites such as deserts and shrublands, regardless of the degree of fire-induced vegetation mortality, compared to relative metrics such as the relativized delta normalized burn ratio (RdNBR) [63] or relativized burn ratio (RBR) [37]. Indeed, we explored how RBR [37] varied along gradients of AET and WD; shapes of the relationships were nearly identical to those we reported for dNBR, but the strengths of the relationships were weaker. In summary, although fire severity has ecological significance beyond what can be inferred from dNBR, we used dNBR as a convenient and standardized way to assess fire severity.

Several additional factors should be considered when interpreting our results. First, although we focused our study on wilderness and national parks, fire regimes are not entirely natural in those areas. Fire exclusion does occur, which reduces fire activity [65] and increases the potential for high severity fire in some forest types [5]. This factor may blur the relationship between fire and climate. Second, the spatial resolution of our analysis (i.e., size of hexels) does not allow us to adequately represent the substantial variability in climate and fire regime characteristics within each hexel [52]. Third, though we used AET and WD as proxies for fuel amount and fuel moisture, respectively, there may be some ecosystems where these proxies are less suitable. For example, AET may poorly represent fuel amount in ecosystems with high rates of decomposition (tropics), or those that experience frequent fire. Finally, because wilderness areas and national parks are often located at high elevations (e.g., at the tops of mountain ranges), the climatic conditions within hexels included in this study are not necessarily representative of the ecoregions as a whole [66]; for example, many of the hexels in the Pacific Northwest and Sierra Nevada and Klamath ecoregions have a relatively high proportion of unproductive alpine environments, and they hence have lower AET than the ecoregion on average.

## Conclusion

A growing body of literature on pyrogeography points to relatively simple energetic controls on fire occurrence [58]: fires require fuel and that fuel needs to be dry enough to burn. To the extent that AET and WD are suitable proxies for fuel amount and fuel moisture, respectively, we found that fire activity across the western US is clearly limited by lack of available fuel and fuel moisture conditions. Furthermore, we found that fire severity is positively related to both fuel amount and fuel moisture. To our knowledge, this is the first broad-scale characterization of fire activity *and* severity along physical environmental gradients. A pyrogeographic perspective that includes both fire activity and severity provides an enhanced view of contemporary fire regimes that complements existing classifications of fire activity. As such, the concurrent characterization of fire activity and severity was effective in distinguishing contemporary fire regime properties of most ecoregions in the western US. The explicit links between fire activity and severity with physical environmental gradients provides a necessary first step for generating multivariable statistical models to produce an empirically based fire regime map for the western US. Such models will potentially enable researchers to predict the geographic distribution of future fire regime characteristics based on gridded spatial data representing future climate scenarios. Consequently, our framework should advance the study of climate-mediated impacts on fire regimes, because, although numerous studies have predicted changes in fire activity due to climate change (e.g., [67]), none have yet examined how fire severity is predicted to change.
